# BAP31-Mediated miR-206/133b Cluster Promotes Transendothelial Migration and Metastasis of Colorectal Cancer

**DOI:** 10.3390/ijms242316740

**Published:** 2023-11-25

**Authors:** Qi Zhang, Changli Wang, Yufei Wu, Jingjing Liu, Tianyi Wang, Bing Wang

**Affiliations:** Institute of Biochemistry and Molecular Biology, College of Life and Health Sciences, Northeastern University, Shenyang 110819, China; 1810067@stu.neu.edu.cn (Q.Z.); 1710070@stu.neu.edu.cn (C.W.); 2301528@stu.neu.edu.cn (Y.W.); 1910069@stu.neu.edu.cn (J.L.)

**Keywords:** BAP31, the miR-206/133b cluster, transendothelial migration, HOXD10

## Abstract

Dysregulated B cell receptor-associated protein 31 (BAP31) plays a crucial role in tumor progression. This study aimed to investigate the functions and molecular mechanism of BAP31 on the miR-206/133b cluster in colorectal cancer (CRC). qPCR was conducted to detect miRNA and mRNA levels in tissues and cells. Western blot assays were used to assess the levels of biomarkers and targets, as well as the levels of BAP31 and HOXD10. Wound healing, coculture and transwell assays were conducted to assess the transendothelial migration abilities of CRC cells. A luciferase assay was employed to assess miRNA binding effects on targets, as well as the initiating transcription effect of genomic fragments. Tumor growth and lung metastatic models were established through an in vivo animal study. BAP31 overexpression in CRC cells led to a reduction in the expression of the miR-206/133b cluster. The expression of the miR-206/133b cluster was correlated with the transendothelial migration capability of CRC cells. The miR-206/133b cluster was found to directly regulate cell division cycle 42 (CDC42) and actin-related protein 2/3 complex subunit 5 (ARPC5) in the tight junction pathway (hsa04530). Moreover, a potential transcription regulator of the miR-206/133b cluster was also found to be Homeobox D10 (HOXD10). We further elucidated the molecular mechanisms and functional mechanisms of BAP31’s regulatory role in the expression levels of the miR-206/133b cluster by inhibiting HOXD10 translocation from the cytoplasm to the nucleus. In conclusion, this study provides valuable insights into how BAP31 regulates the transcription of the miR-206/133b cluster and how BAP31-related lung metastases arise in CRC.

## 1. Introduction

Previous studies have indicated that B cell receptor-associated protein 31 (BAP31), a multifunctional integral protein located on the endoplasmic reticulum (ER) membrane, plays a regulatory role in modulating various molecules and participating in multiple cellular processes in tumor cells [1,2,3,4,5]. In colorectal cancer (CRC), the expression of BAP31 was significantly associated with advanced clinical stages, particularly in stage II and III cases [6]. Additionally, BAP31 expression was significantly higher in metastatic CRC tissues compared to primary CRC tissues [7]. Although significant progress has been made in understanding the downstream mediators of BAP31 function, the precise molecular mechanism responsible for dysregulated BAP31 in CRC cells remains largely uncertain.

miRNAs, which are approximately 22 nucleotides long, regulate gene expression in animals and plants by cleaving or repressing the translation of target mRNAs depending on the level of similarity to their target sequence. Multiple studies have provided evidence supporting the role of miRNAs in tumor progression [8,9,10]. Comprehensive analyses of miRNA expression patterns have been conducted in various human cancers, revealing distinct patterns [11,12,13,14,15]. Some miRNA-encoding genes are located in specific regions of the genome and are called miRNA clusters. Within an miRNA cluster, miRNA-encoding genes can be regulated by a shared regulatory unit and are co-expressed [16,17]. Members of an miRNA cluster either have shared targets or target distinct genes associated with specific pathways [16]. The miR-17-92 cluster, located on chromosome 13q31.3, is a well-known miRNA cluster and is classified as an oncogene that collaborates with c-myc to promote tumor development [18]. Furthermore, substantial advancements have been achieved in comprehending the mechanism of miRNA biosynthesis. RNA polymerase II initially synthesizes longer precursor transcripts with hairpin structures, subsequently undergoing processing by Drosha and Dicer ribonucleases to generate mature miRNAs [19]. However, the specific regulatory mechanisms that affect miRNA performance during the progression of CRC remain largely unclear. As we are aware, miRNA coding sequences are dispersed throughout the entire genome and are typically classified according to their location: intergenic, intronic, and within host genes. Intergenic miRNAs are regulated by their own promoter, while host gene-derived miRNAs originate from either the exon or intron of the lncRNA (host gene), sharing a promoter with their host genes. Intronic miRNAs can have their own independent promoter or share a promoter with the host gene [20,21,22]. The transcriptional regulation of miRNA expression is hypothesized to be orchestrated by a group of cellular transcription factors that interact with the promoter region, akin to protein-coding genes. Several studies have experimentally validated the core promoters of miRNAs, primarily predicted by bioinformatics algorithms that target the upstream sequences of their precursors [23,24,25,26].

Given our long-standing interest in understanding the regulation of miRNA by the BAP31 gene in CRC [27], we sought to explore a potential regulatory mechanism in which BAP31 may participate in miRNA expression to regulate tumor-related genes. This study aimed to examine the influence of BAP31 on the expression of the miR-206/133b cluster in CRC. The expression of the miR-206/133b cluster, regulated by BAP31, plays a role in the transendothelial migration of CRC cells, which is dependent on the transcription factor Homeobox D10 (HOXD10). Disrupting HOXD10 in this regulatory axis significantly mitigated the phenotypic effects of transendothelial migration and metastasis in BAP31-overexpressing CRC cells, providing further insights into the molecular mechanisms that drive the progression of BAP31-associated tumors.

## 2. Results

### 2.1. BAP31-Mediated Regulation of the miR-206/133b Cluster

**Figure 1:**
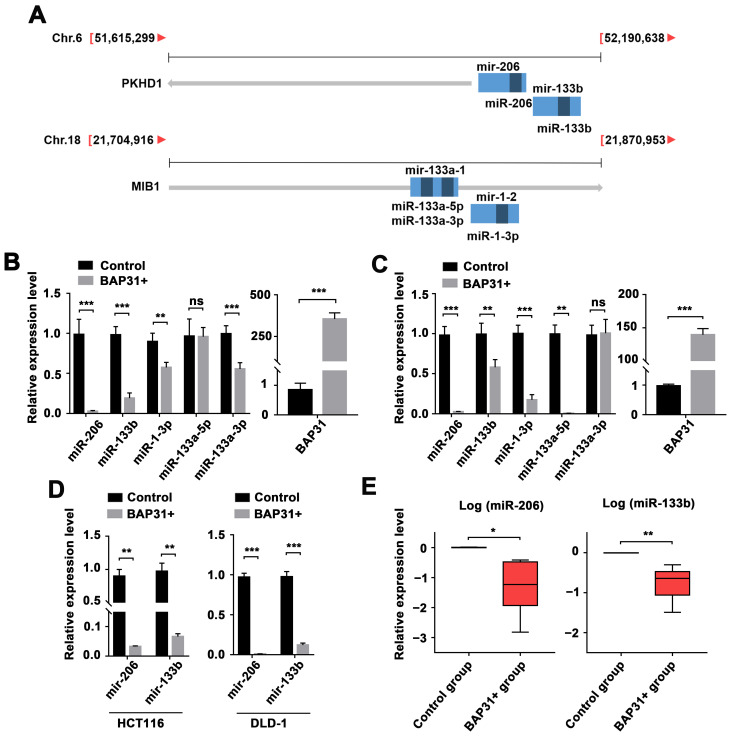
Regulation of the miR-206/133b cluster is associated with BAP31 expression levels in CRC cells and tumor tissues. (**A**) Schematic diagram demonstrating the locations of the miR-206/133b and miR-133a-1/1-2 clusters. The expressed sequence tag transcripts were identified in the UCSC database. Light blocks indicate the position of miRNA precursors, and dark blocks indicate the position of miRNA matures. (**B**,**C**) qPCR analysis of the expression of the miR-206/133b cluster and the miR-133a-1/1-2 cluster in HCT116 (**B**) and DLD-1 (**C**) with BAP31 overexpression. (**D**) qPCR analysis of expression of miR-206/133b cluster precursors in HCT116 and DLD-1 cells. (**E**) qPCR analysis of the expression of miR-206 and miR-133b in CRC xenograft tumors. Data are represented as the mean ± SD of three independent experiments. * *p* < 0.05, ** *p* < 0.01, *** *p* < 0.001, and ns indicates non-significance.

In our previous study, we documented the regulatory role of BAP31 in exosomal miRNA expression [27]. However, the mechanism by which BAP31 regulates miRNA expression remains unclear due to its nature as a multi-pass transmembrane protein of the endoplasmic reticulum. To further investigate this regulation, we analyzed the effect of BAP31 on miRNA expression profiles using miRNA sequencing data (BioProject ID: PRJNA884845). We employed a modified global normalization approach [28], based on the method provided by Lianchuan Biotechnology (LC Sciences, Houston, TX, USA), to correct copy numbers among different samples. We identified four specific miRNAs, located within two miRNA clusters (miR-206/133b and miR-133a-1/1-2), that exhibited statistically significant changes. These miRNA clusters were, respectively, encoded by polycistrons located on Chromosome 6 and Chromosome 18, as depicted in Figure 1A and Table S1.

Since these miRNAs were detected in exosomes, we sought to explore their regulation patterns in cellular contexts. Accordingly, we established three CRC cell lines with BAP31 overexpression, as confirmed via qPCR analysis. To evaluate the abundance of miRNAs, we compared these cell lines to normal CRC cells. The results showed differential expressions of the genomically clustered miR-206 and miR-133b. Additionally, miR-133a-5p, miR-133a-5p, and miR-1-3p, which are also part of the same genomic cluster, displayed distinct differences. Notably, we consistently observed downregulation of miR-206 and miR-133b when BAP31 was overexpressed (Figure 1B,C and Figure S1A). Furthermore, we synthesized primers specific to these miRNA precursors (pre-miRNA) and observed downregulation of both mir-206 (miR-206 precursor) and mir-133b (miR-133b precursor) in CRC cells overexpressing BAP31 (Figure 1D and Figure S1A).

To investigate the expression of miRNA precursors and matures in response to BAP31 overexpression, we injected BAP31-overexpressing HCT116 cells into the back flank of nude mice. The xenograft tumors from this experiment showed detectable levels of both miRNA precursors and matures. Importantly, tumor samples with BAP31 overexpression exhibited lower expression of both precursors and matures compared with control samples. Additionally, the expression of both precursors and matures showed an inverse correlation with BAP31 levels in these tumor tissues (Figure 1E and Figure S1B–D). The findings strongly indicate that amplification of the BAP31 gene led to a decrease in both miR-206/133b cluster precursor and mature forms.

### 2.2. Manipulation of the miR-206/133b Cluster on CRC Cell Transendothelial Migration

**Figure 2:**
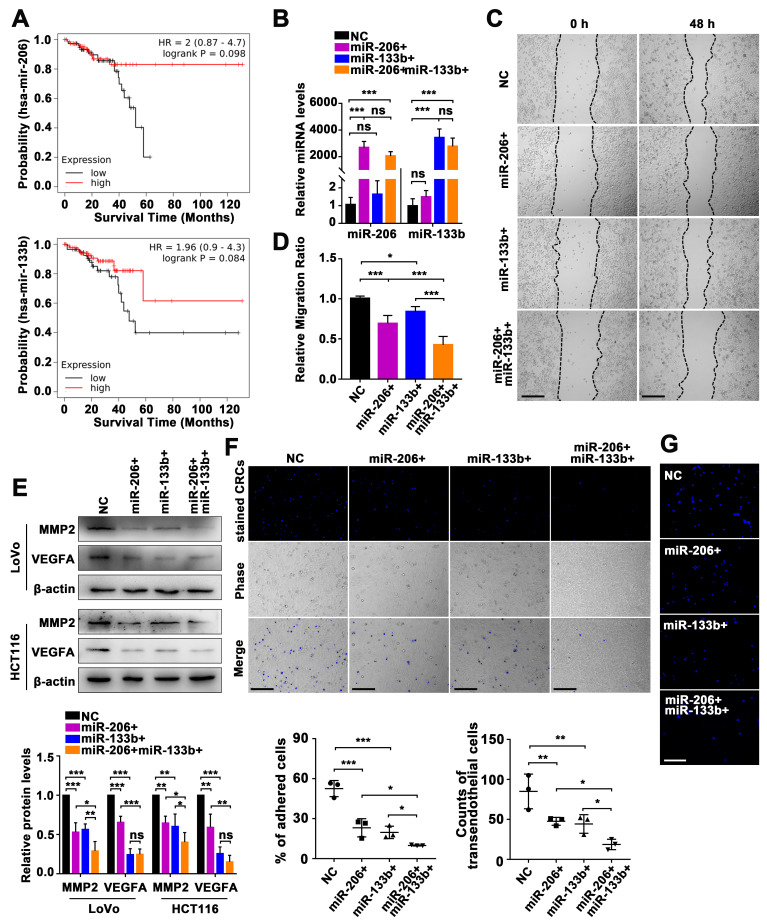
Manipulation of the miR-206/133b cluster affects transendothelial cell migration in CRC cells. (**A**) Kaplan−Meier curves demonstrate the overall survival of patients with rectal adenocarcinoma for mir-206 and mir-133b cases selected with high expression versus low expression cases. (**B**) qPCR analysis of the expression of miR-206 and miR-133b in LoVo cells with transfection of miRNA mimics. (**C**,**D**) Wound healing assays demonstrate the effect of miR-206 and miR-133b on enhancing the migration effect of LoVo cells (Scale bar, 100 μm). (**E**) Western blot analysis of related protein expression reflecting the effect of miR-206 and miR-133b in LoVo cells and HCT116 cells. (**F**) Adhesion assays analyzed the effect of miR-206 and miR-133b in LoVo cells. The representative microscopic images demonstrate the phase contrast of Hoechst33342-stained LoVo cells (top panel) remaining on the layer of HUVECs (middle panel) after multiple washes (Scale bar, 100 µm). (**G**) Transendothelial migration assays demonstrate the effect of miR-206 and miR-133b in LoVo cells. The representative microscopic images demonstrate the phase contrast of the Hoechst33342-stained LoVo cells (blue) during transmigration through an endothelial barrier (Scale bar, 50 µm). Data are represented as the mean ± SD of three independent experiments. * *p* < 0.05, ** *p* < 0.01, *** *p* < 0.001, and ns indicates non-significance.

To evaluate the functional consequences of the miR-206/133b cluster in CRC, we initially examined its clinical efficacy as a potential diagnostic and prognostic biomarker for CRC. We utilized the online public Kaplan–Meier Plotter database [29] to investigate the association between the expression levels of miRNA precursors (mir-206 and mir-133b) and the overall survival of patients with rectal adenocarcinoma. The results reveal a negative correlation between the expression levels of these miRNA precursors and overall survival in this patient population (Figure 2A). Previous studies showed a significant downregulation of miR-206 and miR-133b in CRC tissues, indicating their tumor-suppressive role in CRC progression [30,31,32,33]. To assess the impact of ectopic miR-206 and/or 133b on CRC progression, analysis of the RNA copy number per cell line for miR-206 and miR-133b showed low abundance in LoVo cells, but high abundance in HCT116 cells (Figure S2A,B). LoVo cells were transiently transfected with miR-206 mimics and/or miR-133b mimics. Subsequent qPCR analysis indicated a significant increase in the expression levels of both miR-206 and miR-133b (Figure 2B). Wound healing assays were then conducted to evaluate the effect of the miR-206/133b cluster on migration response. The results indicate that LoVo cells expressing high levels of miR-206 and miR-133b demonstrated delayed cell migration. Additionally, LoVo cells with a concurrent upregulation of miR-206 and miR-133b displayed even lower migration rates (Figure 2C,D).

Subsequent Western blotting analysis showed distinct modulation patterns of ZO-1, E-cadherin, N-cadherin, CD133, CD44, and SOX2 expression by miR-206 and miR-133b levels (Figure S2C). CRC cells with high expressions of miR-206 and miR-133b exhibited the downregulation of MMP2 and VEGFA protein levels, indicating a potential influence on the tumor microenvironment matrix (Figure 2E). To investigate the effect of the miR-206/133b cluster on transendothelial migration, we performed co-cultures of Hoechst33342-stained CRC cells and endothelial cells (ECs) to observe the adherence of CRC cells to ECs. Our fluorescence microscopy observations revealed that LoVo cells stained with Hoechst33342 exhibited decreased adhesion to HUVECs when expressing higher levels of miR-206 and/or miR-133b (Figure 2F). Moreover, LoVo cells expressing high levels of miR-206 and/or miR-133b showed reduced transendothelial migration, compared with control cells. These findings indicate that an increased expression of miR-206 and miR-133b hinders the transendothelial migration of CRC cells (Figure 2G). Additionally, the transfection of HCT116 cells with inhibitors of miR-206 and/or miR-133b resulted in an elevated adhesion of HCT116 cells to HUVECs and enhanced transendothelial migration, as observed through Hoechst33342 staining (Figure S2D,E). These results further indicate that miR-206 and miR-133b had an impact on the adhesion and transendothelial migration capabilities of CRC cells.

### 2.3. Identification of the miR-206/133b Cluster-Targeted Genes in CRC Cells

To investigate the functional mechanism of the miR-206/133b cluster, we used miRDB, TargetScan, and TargetMiner to predict the potential targets of miR-206 and miR-133b [34,35,36]. We analyzed the common potential targets identified employing these miRNA target prediction algorithms. Furthermore, we conducted KEGG pathway analysis (https://www.kegg.jp/ (accessed on 1 March 2023)) to obtain functional annotations for their predicted targets. The outcomes demonstrate that 56 signaling pathways, such as the tight junction, Ras, and Apelin signaling pathways, among others, were enriched in the potential targets of miR-206; however, the three signaling pathways in which the potential targets of miR-133b were enriched were the dopaminergic synapse pathway, the AMPK signaling pathway, and the tight junction pathway. The findings indicate that among the miR-206 and miR-133b target genes, only the tight junction pathway (hsa04530) consistently displayed enrichment with functional annotations. In our current analysis, considering the scoring outcomes from the mentioned algorithms, we further used miRanda to score these target prediction results, as shown in Table S3. We specifically focused on four potential targets within the tight junction pathway for miR-206 and miR-133b: cell division cycle 42 (CDC42), discs large MAGUK scaffold protein 2 (DLG2), actin-related protein 2/3 complex subunit 5 (ARPC5), and protein phosphatase 2 catalytic subunit beta (PPP2CB).

**Figure 3:**
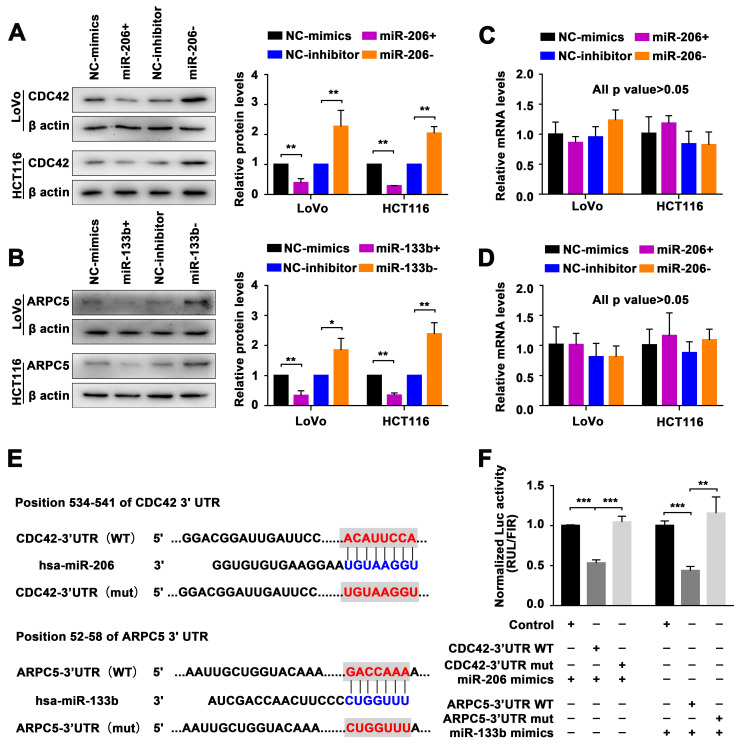
Targets of the miR-206/133b cluster are predicted and identified in CRC cells. (**A**) Western blot analysis of CDC42 protein expression, reflecting the effect of miR-206 in LoVo and HCT116 cells. (**B**) Western blot analysis of ARPC5 protein expression, reflecting the effect of miR-133b in LoVo and HCT116 cells. (**C**) qPCR analysis of CDC42 mRNA expression, reflecting the effect of miR-206 in LoVo and HCT116 cells. (**D**) qPCR analysis of ARPC5 mRNA expression, reflecting the effect of miR-133b in LoVo and HCT116 cells. (**E**) Schematic diagram showing wild-type (WT) and mutation (mut) sequences of CDC42 3′UTR binding sites matching for miR-206, and wild-type (WT) and mutation (mut) sequences of ARPC5 3′UTR binding sites matching for miR-133b. (**F**) Luciferase activity reporter showing the miR-206 binding effect to wild-type (WT) and mutation (mut) sequences of the CDC42 3′-UTR, and the miR-133b binding effect to wild-type (WT) and mutation (mut) sequences of the ARPC5 3′-UTR. Data are represented as the mean ± SD of three independent experiments. * *p* < 0.05, ** *p* < 0.01, and *** *p* < 0.001.

To accurately investigate the molecular function of miRNA, we performed a transient transfection of miRNA mimics to overexpress miR-206 or miR-133b, and miRNA inhibitors to inhibit intrinsic miRNAs in CRC cells (LoVo and HCT116 cells). The protein levels of CDC42, as determined via a Western blot (Figure 3A), showed significant differences when miR-206 was present, but no significant differences were observed at the mRNA level (Figure 3C). Similarly, transfection with either miR-133b mimics or inhibitors resulted in similar changes in the levels of ARPC5 protein and mRNA (Figure 3B,D). These findings clearly demonstrate that miR-181a and miR-181b can have a post-transcriptional regulatory impact on CRC42 and ARPC5.

To further evaluate the validity of these potential targets, we performed a dual luciferase assay to investigate the direct interaction between miRNAs and the 3′-UTR of their putative targets. The dual luciferase reporters were generated by inserting the wild-type (WT) binding sequence of the putative targets at the 3′-end of the Renilla luciferase gene in the psiCHECK-2 luciferase vector. As a control for mutation (mut), the predicted miR-206 or miR-133b response element was converted to its complementary sequence to eliminate potential miRNA binding (Figure 3E). Luciferase reporter assays demonstrate that the construct with the wild-type gene of CDC42 and ARPC5 exhibited significantly reduced luciferase activity in the presence of miR-206 and miR-133b mimics, compared with the control group construct. The presence of the construct with the mutation group eliminated the inhibitory effect of miR-206 and miR-133b mimics (Figure 3F).

### 2.4. Identification of miR-206/133b Cluster Transcription Factors

**Figure 4:**
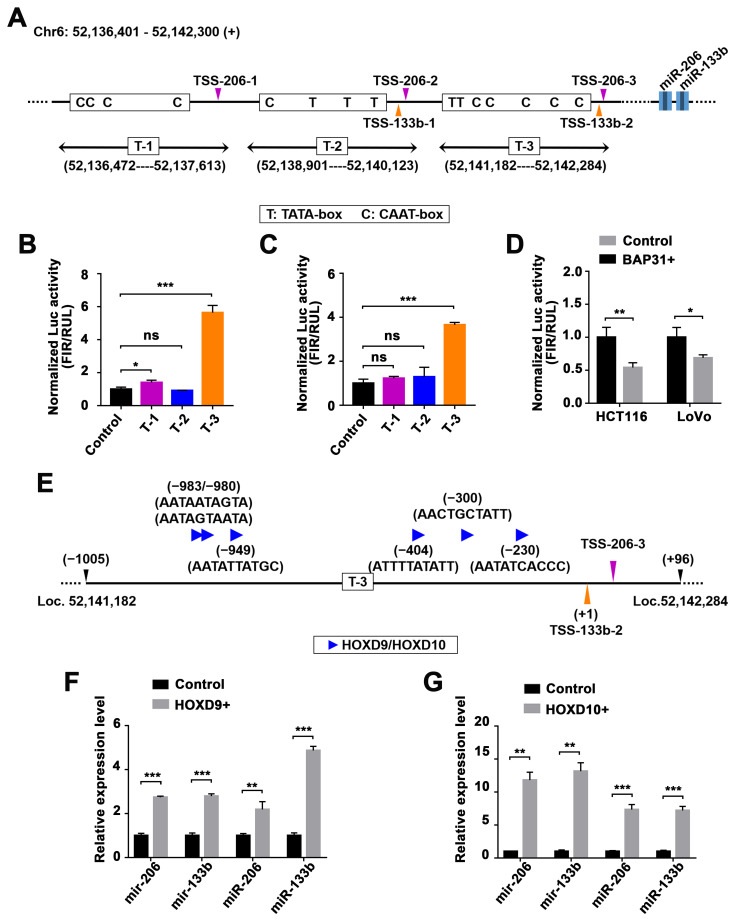
miR-206/133b cluster transcription factors are predicted and identified in CRC cells. (**A**) Schematic diagram demonstrating three regions containing potential TSSs of the miR-206/133b cluster. The cis-acting regulatory DNA elements (TATA-box and CAAT-box) are demonstrated within the motif on the plus strand. Light blocks indicate the position of miRNA precursors, and dark blocks indicate the position of miRNA matures. (**B**,**C**) Luciferase activity reporter showing the initiating transcription of three genomic fragments (T-1, T-2 and T-3 regions) in HCT116 (**B**) and LoVo cells (**C**). (**D**) Luciferase activity reporter showing the initiating transcription of the T-3 region, reflecting BAP31 expression in LoVo and HCT116 cells. (**E**) Schematic diagram demonstrating the HOXD9 and HOXD10 binding sites within the T-3 region motif on the plus strand. (**F**,**G**) qPCR analysis of the expression of miR-206/133b cluster precursors and matures, reflecting the effect of HOXD9 (**F**) and HOXD10 (**G**) in HCT116 cells. Data are represented as the mean ± SD of three independent experiments. * *p* < 0.05, ** *p* < 0.01, *** *p* < 0.001, and ns indicates non-significance.

The expression of the primary cluster transcript was reduced in BAP31-overexpressing CRC cells, indicating that BAP31 may inhibit the transactivation of the cluster in these cells, in line with the decrease in mature miRNA levels. The miR-206/133b cluster is an intergenic cluster known to have its own miRNA promoters [37]. To investigate the transcriptional regulation of the miR-206/133b cluster, we utilized the human genome assembly GRCh38 from the UCSC database (http://genome.ucsc.edu/index.html (accessed on 16 April 2023)) to obtain their upstream sequence. Subsequently, we employed the Promoter2.0 database from DTU Health Tech (https://services.healthtech.dtu.dk/services/Promoter-2.0/ (accessed on 16 April 2023)) to predict the potential transcription start sites (TSSs) of the miR-206/133b cluster. We identified three regions, potentially containing TSSs, within 10 kb upstream of the miR-206 and miR-133b genes by analyzing data on cis-acting regulatory DNA elements and using likelihood scores derived from neural networks and genetic algorithms (Figure 4A). To verify this prediction, we identified cis-acting regulatory DNA elements, such as TATA-boxes, CAAT-boxes, and E-boxes, located upstream of these TSSs (Table S4). Although GC-rich regions were present upstream of these TSSs, no putative CpG islands were predicted.

To further determine these potential TSSs of the miR-206/133b cluster, we isolated and amplified these genomic fragments. The T-1 region spans from 52,136,472 to 52,137,613 in the genomic segment, the T-2 region spans from 52,138,901 to 52,140,123, and the T-3 region spans from 52,141,182 to 52,142,284 on Chromosome 6. These genomic fragments were digested with restriction enzymes and cloned upstream of a vector lacking a Firefly luciferase promoter. The constructs were then transfected into LoVo and HCT116 cells. As shown in Figure 4B,C, the construct containing the T-3 region results in significant increases in luciferase activity compared with the control vector. This suggests that the genomic fragment of the T-3 region has the ability to initiate transcription, while the other two genomic fragments (the T-1 and T-2 regions) do not. We utilized CRC cells that overexpressed BAP31 to further investigate the transcriptional regulation of BAP31 on this fragment. The luciferase activity of CRC cells overexpressing BAP31 was compared to that of the control vector and showed a significant difference in the activity of the genomic fragment containing the T-3 region (Figure 4D).

We then utilized the online software program PROMO (https://alggen.lsi.upc.es/ (accessed on 2 May 2023)) [38] to conduct a search for potential TF-binding sites within the genomic fragment of the T-3 region (Table S5). Multiple binding sequences that match transcription factors Homeobox D9 (HOXD9) and Homeobox D10 (HOXD10) were identified in the T-3 region (Figure 4E). To further determine the involvement of HOXD9 and HOXD10 in the transcriptional activity of the miR-206/133b cluster, we conducted an overexpression experiment in CRC cells. We quantified the levels of miRNA precursors and mature forms using qPCR. The results show that both HOXD9 (Figure 4F) and HOXD10 (Figure 4G) upregulated the expression of miR-206/miR-133b precursors, leading to a corresponding increase in the levels of mature miRNAs. Overall, the transcriptional activation of the miR-206/133b cluster was increased in CRC cells in response to HOXD9 and HOXD10.

### 2.5. BAP31-Mediated Transcriptional Activation of the miR-206/133b Cluster by HOXD10

**Figure 5:**
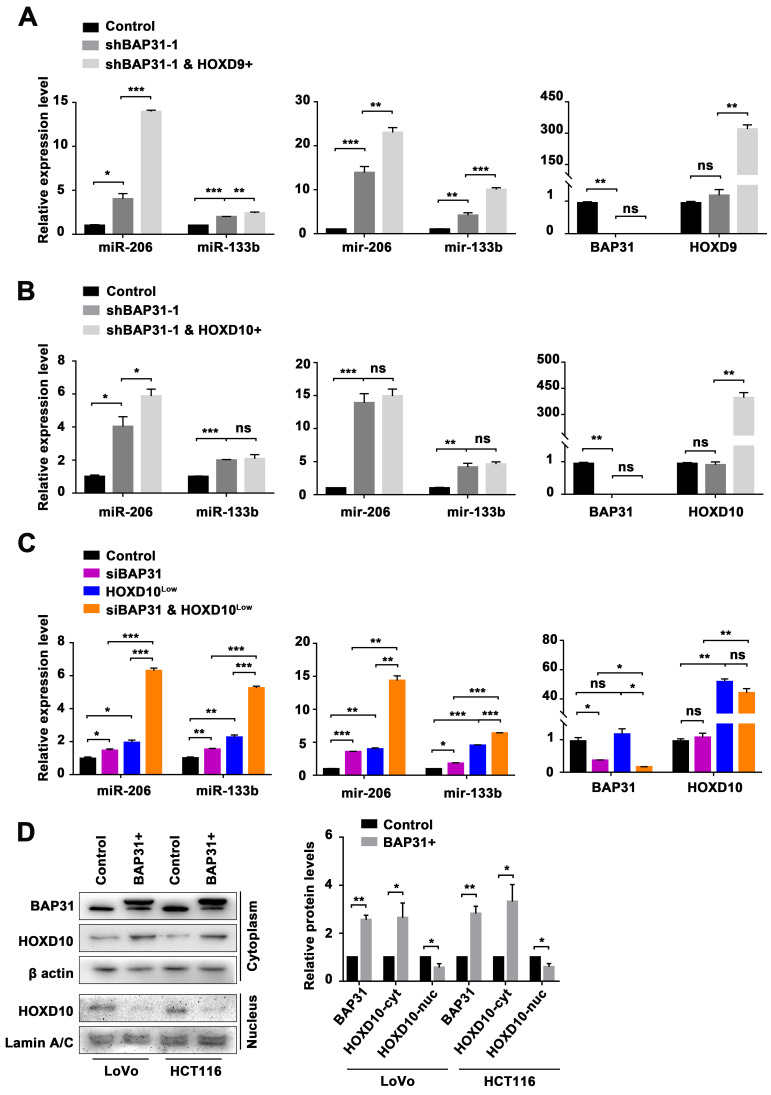
BAP31 manipulates the transcriptional activation of the miR-206/133b cluster by HOXD10. (**A**) qPCR analysis of the expression of miR-206/133b cluster matures and precursors, reflecting the effect of BAP31 and HOXD9 in HCT116 cells. (**B**) qPCR analysis of the expression of miR-206/133b cluster matures and precursors, reflecting the effect of BAP31 and HOXD10 in HCT116 cells. (**C**) qPCR analysis of the expression of miR-206/133b cluster matures and precursors, reflecting the effect of BAP31 and HOXD10 in HCT116 cells. (**D**) Western blot analysis of the expression of HOXD10 gene in the cytoplasm and nucleus fractions, reflecting the effect of BAP31 in HCT116 and LoVo cells. Data are represented as the mean ± SD of three independent experiments. * *p* < 0.05, ** *p* < 0.01, *** *p* < 0.001, and ns indicates non-significance.

We have previously shown that an overexpression of BAP31 resulted in a decrease in the expression of both precursors and matures of the miR-206/133b cluster. Additionally, our results indicate that HOXD9 and HOXD10 promote the expression of both precursors and matures of the miR-206/133b cluster. Accordingly, we propose that BAP31 might exert an influence on the function of transcription factors, HOXD9 or HOXD10, as evidenced by changes in the expression of clustered miRNAs. To investigate the potential role of BAP31 in regulating miRNA transcription via HOXD9 or HOXD10, we conducted experiments using CRC cells that were either overexpressing HOXD9/10 or BAP31. Our results show that both HOXD9 and HOXD10 counteracted the downregulation in the expression of the miR-206/133b cluster caused by BAP31 overexpression (Figure S4A,B). We transfected established stable BAP31 knockdown cell lines (shBAP31-1 and shBAP31-2) with overexpression vectors for HOXD9 or HOXD10. We observed a significant differential expression of both precursors and matures of the miR-206/133b cluster in cells overexpressing HOXD9 (Figure 5A), while there was no significant differential expression in cells overexpressing HOXD10 (Figure 5B). We further validated this finding in another established stable BAP31 knockdown cell line (Figure S4C). Based on the above results, it was hypothesized that upregulating the expression of HOXD10 following BAP31 knockdown could lead to functional redundancy of HOXD10, as evidenced by the lack of a significant difference in the expression of the miR-206/133b cluster. Thus, we utilized CRC cells in which BAP31 was silenced and HOXD10 was overexpressed to examine the expression of their precursor and matures. BAP31 was silenced by transfecting with siBAP31, while HOXD10 was overexpressed by transfecting with HOXD10-overexpressing vectors at different concentrations. In the siBAP31 and HOXD10^low^ group, we observed a significant expression of both precursors and matures of the miR-206/133b cluster compared with the HOXD10^low^ group (achieved by transfecting with a low concentration of HOXD10-overexpressing vectors) (Figure 5C). But the difference in the expression of their precursors and matures was diminished in the siBAP31 and HOXD10^high^ group compared with the HOXD10^high^ group (achieved by transfecting with a high concentration of HOXD10-overexpressing vectors) (Figure S4D). The upregulation of HOXD10 resulted in an increase in both precursors and matures of the miR-206/133b cluster, but the downregulation of BAP31 may have diminished this regulatory effect, resulting in a functional redundancy of HOXD10 (Figure S4E). To further investigate whether BAP31 regulates the transcription of the miR-206/133b cluster through the mediation of HOXD10, we assessed the effect of BAP31 on the translocation of HOXD10 between the nucleus and cytoplasm. Western blotting analysis revealed a significant decrease in the translocation of HOXD10 from the cytoplasm to the nucleus when BAP31 was overexpressed in CRC cells (Figure 5D). The findings indicate that HOXD9 and HOXD10 both counteract the impact of BAP31 on the expression of the miR-206/133b cluster, but BAP31 influences the transcriptional activation of the miR-206/133b cluster by inhibiting the translocation of HOXD10 from the cytoplasm to the nucleus.

### 2.6. Manipulation of the BAP31/HOXD10 Axis in the Tumor Metastasis of CRC

**Figure 6:**
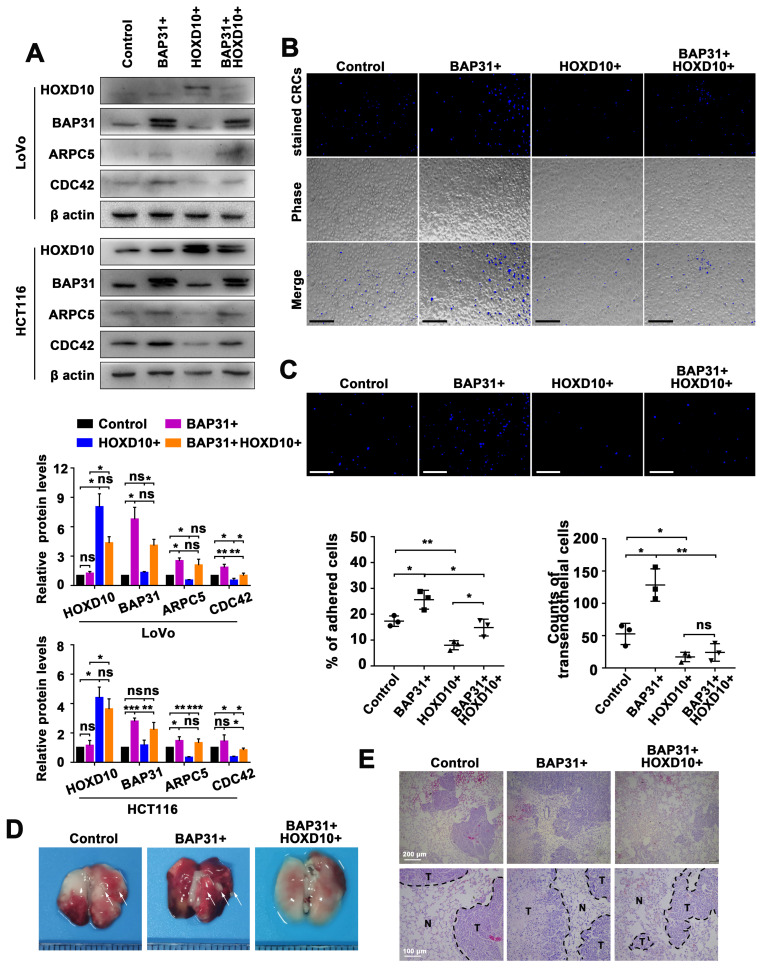
The BAP31/HOXD10 axis manipulates CDC42 and ARPC5 to affect tumor metastasis in vitro and in vivo. (**A**) Western blot analysis of the expression of CDC42 and ARPC5 genes in HCT116 and LoVo cells, reflecting the effect of the BAP31 and HOXD10 genes. (**B**) Adhesion assay results for the effect of BAP31 and HOXD10 in LoVo cells. The representative microscopic images demonstrate the phase contrast of Hoechst33342-stained LoVo cells (top panel) remaining on the layer of HUVECs after multiple washes (Scale bar, 100 µm). (**C**) Transendothelial migration assay results for the effect of BAP31 and HOXD10 in LoVo cells. The representative microscopic images demonstrate the phase contrast of the Hoechst33342-stained LoVo cells (blue) during transmigration through an endothelial barrier (Scale bar, 50 µm). (**D**) Colorectal cancer lung metastasis regulated by BAP31 and HOXD10 expression in nude mice. The arrowheads indicate visible metastatic tumors. (**E**) Representative H&E-stained lung sections prepared from recipient mice bearing CRC cells regulated by BAP31 and HOXD10 expression. N, normal lung tissue; T, metastatic tumor. Data are represented as the mean ± SD of three independent experiments. * *p* < 0.05, ** *p* < 0.01, *** *p* < 0.001, and ns indicates non-significance.

Our findings support a theoretical framework where HOXD10 acts as a limiting factor for the BAP31-mediated transcription of the miR-206/133b cluster. To further validate the impact of this regulatory axis on the transendothelial migration of CRC, we initially examined the effect of the BAP31/HOXD10 axis on their targets of the miR-206/133b cluster in CRC cells that overexpressed BAP31 or HOXD10. The overexpression of BAP31 and HOXD10 resulted in significant differences in the protein expression of CDC42 and ARPC5. Additionally, HOXD10 mitigated the upregulation of their targets caused by BAP31 overexpression (Figure 6A). We subsequently conducted co-culture experiments to visualize the adhesion of CRC cells to ECs. The results demonstrate that viable Hoechst33342-stained CRC cells with overexpressing BAP31 exhibited increased adhesion to ECs, whereas stained CRC cells with overexpressing HOXD10 exhibited decreased adhesion to ECs. Additionally, HOXD10 was observed to counteract the increased adhesion effect of stained CRCs with overexpressing BAP31 to ECs (Figure 6B). In line with the adhesion experiment results, the high BAP31 group exhibited a greater number of transendothelial migrated CRC cells, while the high HOXD10 group showed a lower number of transendothelial migrated CRC cells, compared with their respective control cells. Moreover, HOXD10 impeded BAP31-mediated transendothelial migration (Figure 6C).

Hematogenous metastasis, a common form of metastasis in humans, was induced by intravenously injecting cells through the tail vein, resulting in the development of lung metastases [39]. Nude mice were used as an animal model to investigate the impact of HOXD10 on BAP31-mediated CRC lung metastasis in vivo. The mice were intravenously injected (i.v.) with CRC cells overexpressing BAP31 and HOXD10. Metastatic tumors were induced in BALB/c nude mice, which were then sacrificed at week 4. The lungs were subsequently removed for examination (Figure 6D). HE staining of lung tissues revealed that the carcinoma cells exhibited hyperchromatic nuclei with irregular nuclear membranes and prominent nuclei, in contrast to adjacent normal tissues (Figure 6E). These results further support the significant reduction in the incidence of BAP31 overexpression-induced CRC lung metastasis by HOXD10 overexpression.

## 3. Discussion

According to previous reports on CRC, the expression of BAP31 was found to be correlated with advanced clinical stages, particularly in stage II and III cases [6]. Additionally, BAP31 showed higher expression levels in liver metastatic CRC tissues when compared with primary CRC tissues [7]. Although there have been extensive studies on the downstream mediators of BAP31 function that have significantly improved our understanding, the exact molecular mechanism of dysregulating BAP31 in CRC cells remains largely unknown. Our research has primarily focused on studying how BAP31 regulates the progression of CRC. In a previous report, we highlighted that CRC cells overexpressing BAP31 release exosomes that contain a substantial quantity of miRNAs [27]. However, the transcriptional regulation of miRNAs in response to BAP31 remains undefined based on our previous investigations. Therefore, the primary objective of this study was to further analyze the miRNA/miRNAs modulated by BAP31 expression, in order to provide detailed evidence supporting the regulatory influence of BAP31 on miRNAs. Our research confirmed and extended the evidence that the genomic clustered miR-206 and miR-133b (miR-206/133b cluster) were reduced in BAP31-overexpressing CRC cells (Figure 1). Furthermore, we pre-analyzed the expression of miRNAs in CRC cell lines to explore the effect of variations in miRNA expression on observable phenotype. We found that the expression of miR-206 and miR-133b varied in CRC cell lines. Further explanation is needed for the causes of these variations. Victor Ambros suggested that some microRNAs may have redundant functions compared with other miRNAs with similar sequences [40], which could explain the observed differences. However, compared with normal cells (FHC), lower expression levels of both miRNAs were observed in CRC cells. This clarified the observed variation in miRNA expression across CRC cells, despite the notion that miRNA expression is associated with cancer phenotypes.

We have identified the role of the miR-206/133b cluster in the transendothelial adhesion and migration of CRC cells (Figure 2). Various roles of miRNA genes have been identified, including the regulation of developmental timing, spatial patterning of cell fates, and cellular and organismal physiology. However, we still do not know all the parameters that define a functional miRNA–target interaction. Therefore, informatics approaches can be designed to identify potential interactions, followed by validation using biochemical evidence and genetic epistasis. In this study, we conducted KEGG pathway analysis to obtain functional annotations for the predicted targets. The results consistently showed an enrichment of functional annotations related to the tight junction pathway (hsa04530) for both miR-206 and miR-133b target genes. Furthermore, depending on how they interact with the mRNA ribonucleoprotein and affect its structure and composition, miRNAs have the potential to regulate gene expression in either a positive or negative way. Translation repression can also result from miRNAs binding to particular regulatory regions in the mRNA 3′UTR. Some miRNAs may even control the stability or localization of mRNA. Rather than affecting the mRNA levels of their target genes, our findings indicate that miR-206 and miR-133b had a significant impact on the protein expression of CDC42 and ARPC5. From this finding, it is evident that both miRNAs control the post-transcriptional suppression of CDC42 and ARPC5. Here, we further provide detailed evidence to supplement and expand the significant role of the miR-206/133b cluster in the adhesion and migration phenotype.

To the best of our knowledge, longer precursor transcripts (pri-miRNAs) with hairpin structures are transcribed initially by RNA polymerase II. Subsequently, miRNA precursors are processed further to form precursor miRNAs (pre-miRNAs) through the action of nuclease Drosha. Exprotin-5 transports these pre-miRNAs into the cytoplasm, where they are cleaved by the Dicer enzyme to generate mature miRNAs [41,42]. This study focused on the intergenic miR-206/133b cluster, which are transcribed under the regulation of an independent promoter region [43]. Generally, the transcription start site (TSS) is located at the 5’ end of pri-miRNA, and the sequence preceding the TSS is referred to as the promoter. Intergenic miRNAs are expected to have pri-miRNAs located within 10 kb and exhibit a conserved pattern similar to the promoter of protein-coding genes. Although our understanding of miRNA expression regulation is limited, it has been proposed that this process is orchestrated by a cellular pool of transcription factors that interact with the promoter region. For instance, c-MYC and E2F proteins were shown to regulate the transcription of the miR17-92 polycistron [44,45,46]. In this study, we investigated three genomic fragments located within a 10 kb nucleotide range upstream of the miR-206/133b cluster. The T-1 region spans from 52,136,472 to 52,137,613 on Chromosome 6, the T-2 region spans from 52,138,901 to 52,140,123, and the T-3 region spans from 52,141,182 to 52,142,284. These regions were identified based on transcription start sites (TSSs) predicted in the Promoter2.0 database from DTU Health Tech. Significant differences were observed in the T-3 region of BAP31-overexpressing CRC cells (Figure 4B,C). Through analysis of the T-3 region, potential transcription factor (TF)-binding sites, specifically HOXD9 and HOXD10, were predicted. We attempted to investigate the interaction between BAP31 and HOXD9/HOXD10 through the utilization of the STRING database (https://string-db.org/ (accessed on 5 May 2023)) [47] (Figure S3), as well as Western blotting and qPCR assays (not displayed). But our investigation did not yield any evidence supporting the regulation of HOXD9 or HOXD10 expression by BAP31. However, the downregulation of the BAP31 gene resulted in an observed increase in the miR-206/133b cluster in CRCs, which was found to be dependent on HOXD10 (Figure 5). Disrupting this regulatory axis with HOXD10 significantly alleviated the phenotype of BAP31 overexpression-induced CRC lung metastasis, providing further insight into the molecular mechanism underlying BAP31-involved cancer progression (Figure 6). However, additional clarification is needed to fully understand the precise mechanism by which BAP31 interacts with HOXD10 to control the transcription of the miR-206/133b cluster.

**Figure 7:**
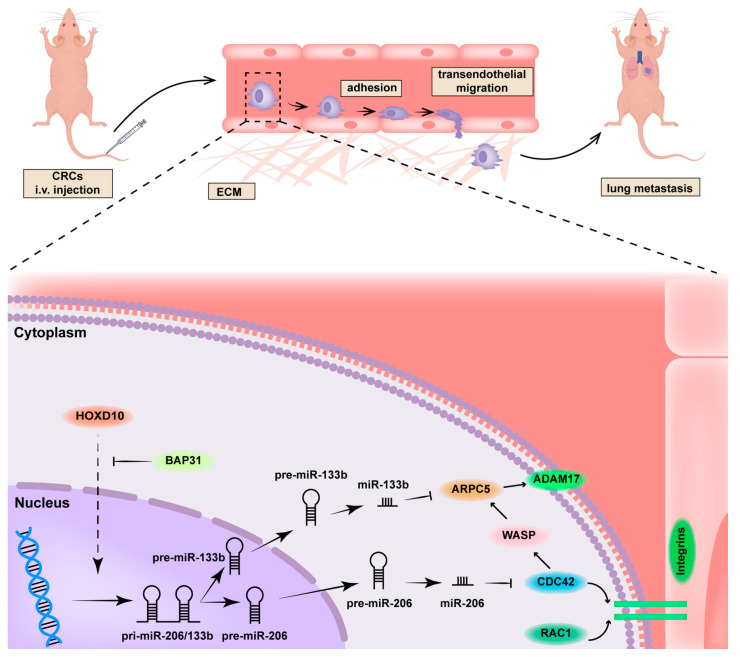
Schematic diagram demonstrating that BAP31 regulates the miR-206/133b cluster through HOXD10, promoting transendothelial migration and lung metastasis in CRC.

Overall, there are still some questions in this study that require further investigation. It is necessary to explore the detailed mechanism through which BAP31 controls HOXD10. A more detailed explanation is needed to understand the variations in miRNA expression in different tumor cells. Our study has, however, uncovered a regulatory pathway by which BAP31 regulates the transcriptional control of the miR-206/133b cluster. This mechanism contributes to the enhancement of transendothelial migration and lung metastasis in colorectal cancer. Building upon previous studies [48,49], we have elucidated a regulatory pathway, as illustrated in Figure 7.

Our conclusions are as follows: (i) The overexpression of BAP31 leads to a decrease in both precursors and matures of the miR-206/133b cluster in CRC cells; (ii) miR-206 directly targets CDC42, while miR-133b targets ARPC5, facilitating the transendothelial migration of CRC cells; (iii) The regulation of the miR-206/133b cluster in BAP31-overexpressing CRC cells depends on the translocation of HOXD10 from the cytoplasm to the nucleus, and disrupting this regulatory axis with HOXD10 significantly weakens the phenotype of transendothelial migration and metastasis in BAP31-overexpressing induced CRC cells. These findings provide valuable insights for a comprehensive investigation of the BAP31-regulated transcription of the miR-206/133b cluster and a thorough exploration of its molecular mechanism.

## 4. Materials and Methods

### 4.1. Cell Culture

Human umbilical vein endothelial cells (HUVEC), human CRC cells (HCT116, DLD-1, SW480, HT29 and LoVo), and HEK-293T cells were cultured in our laboratory. HUVEC, HCT116, DLD-1, SW480, HT29, and HEK-293T cells were cultured in a DMEM medium (Gibco, New York, NY, USA), and LoVo cells were cultured in an RPMI 1640 medium (Gibco). The cells used are listed in Table S6. Both media were supplemented with 10% FBS, 100 U/mL penicillin, and 0.1 mg/mL streptomycin. The cells were maintained in a humidified atmosphere of 5% CO_2_ at 37 °C.

CRC cells were transfected with adenovirus plasmids (pcDNA3.1(-)-BAP31-Flag, pcDNA3.1(-)-HOXD9-HA, and pcDNA3.1(-)-HOXD10-HA) and miRNA mimics/inhibitor (GenePharma, Shanghai, China) using Neofect™ transfection reagent (Neofect biotech, Beijing, China). Stable cell lines were established by transducing CRC cells with lentivirus produced by HEK-293T cells according to a standardized protocol [1]. Expression levels of the genes were detected using qPCR or Western blotting analyses.

### 4.2. RNA Analysis

qPCR was used to determine miRNA and mRNA in CRC cells. For the miRNA matures, we employed the stem ring method to precisely complement and bind to the target miRNA. We then used the RT reaction to obtain extended miRNA cDNA, and subsequently used qPCR to measure the abundance of target miRNA. For miRNA precursors and mRNA, we reverse-transcribed the miRNA precursors and mRNA into cDNA using random primers, and then used qPCR to determine the abundance of miRNA precursors.

Total RNA was extracted according to the protocol provided by the Trizol Reagent (Sigma-Aldrich, Darmstadt, Germany). To quantify miRNA, 2 μg of total RNA was reverse transcribed into cDNA using miRNA-specific stem-loop primers. To quantify mRNA, cDNA was synthesized from 2 μg of total RNA using oligo (dT) primers in a reverse transcription kit (Takara, Dalian, China) as per the manufacturer’s recommendation. qPCR was conducted using the SYBR Green PCR Master Mix (Vazyme, Nanjing, China) on the Bio-RAD CFX96 Real-Time PCR System with a final volume of 20 μL. The expression of miRNA was normalized to U6, while the expression of mRNAs was normalized to GAPDH. The data are presented as relative quantification calculated using 2^−ΔΔCt^.

The primers used (GENEWIZ, Suzhou, China) are listed In Table S2.

### 4.3. Western Blot Analysis

Cell or tissue samples were lysed using the RIPA Lysis Buffer (Beyotime, Shanghai, China), supplemented with a Protease Inhibitor Cocktail (Abcam, Cambridge, UK), and quantified using the BCA Protein Assay Kit (Beyotime). The protein samples were separated on a 6–15% SDS-PAGE gel and transferred to PVDF membranes (ThermoFisher, Waltham, MA, USA). After blocking with 5% non-fat milk, the membranes were incubated overnight at 4 °C with primary antibodies, followed by incubation with the appropriate secondary antibody. Finally, the blots were developed using an ECL detection kit (ThermoFisher Scientific) in the Bio-Rad ChemiDoc™ imaging system. The ratios of target protein band intensities relative to that of the housekeeping protein, which reflect changes in expression levels, were calculated using ImageJ software (ImageJ 1.52a, NIH, Bethesda, MD, USA).

The antibodies used are listed in Table S7.

### 4.4. Wound Healing Assay

CRC cells were transfected with miRNA mimics as per the experimental design. The treated CRC cells were plated and then scraped away using a pipette tip. After a 48 h incubation period, images were captured using a microscope. Migrated cells were observed in four randomly selected fields. The relative migration ratios, indicating the extent of migration changes, were calculated using ImageJ software (NIH).

### 4.5. In Vitro Adhesion and Transendothelial Migration Assay

To examine the effect of the miR-206/133b cluster on adhesion ability, CRC cells were transfected with miRNA mimics/inhibitor according to the experimental design. HUVEC cells were seeded in a 24-well plate. After 24 h, the treated CRC cells were collected and stained with Hoechst 33342 (Beyotime). The labeled CRC cells were then added to the HUVEC cells at a concentration of 10^5^ cells/well and co-incubated for 30 min. Non-adherent CRC cells were gently washed with phosphate-buffered saline (PBS) to remove them. The fluorescence signals of the adherent cells were observed using a fluorescence microscope (Leica, Weztlar, Germany).

To examine the effect of the miR-206/133b cluster on transendothelial migration ability, CRC cells were transfected with miRNA mimics/inhibitors according to the experimental design. HUVEC cells were seeded onto top chamber inserts with 8 μm pores (Corning, New York, NY, USA). After 24 h, the treated CRC cells were collected and stained with Hoechst 33342 (Beyotime). The labeled CRC cells were added to HUVECs at a concentration of 10^5^ cells per well and co-incubated for 24 h. Cotton swabs were used to wipe off the non-migrated CRC cells remaining on the upper side of the membrane. The fluorescence microscope (Leica) was used to observe the fluorescent signals of the transendothelial migrated cells.

### 4.6. Luciferase Reporter Assay

The luciferase reporter was used to determine the binding effect of miRNA on potential targets and the transcription initiation effect of genomic fragments. For miRNA-target interaction, Renilla luciferase was suppressed as a result of miRNA-target interactions, pairing with specific mRNA 3′UTR regulatory elements, and translation inhibition. For the transcription initiation effect of genomic fragments, Firefly luciferase was activated to detect the transcription start sites and related elements present in the genomic fragments that were found to have an effect on transcription initiation.

To examine the binding effect of miRNA on potential targets, we constructed psiCHECK-2 vectors that contained the wild-type or mutated 3′UTR sequences of CDC42 or ARPC5. We co-transfected these vectors along with miRNA mimics into cells. The Dual-Luciferase Reporter Gene Assay Kit (Beyotime) was used to measure the activities of Firefly and Renilla luciferase. The relative luciferase activity was calculated by normalizing the values of Renilla/Firefly. To examine the transcription initiation effect of genomic fragments, the T-1, T-2, and T-3 regions were inserted into a luciferase reporter plasmid (PGL420 vector). These constructs, along with a Renilla luciferase plasmid (pRL-SV40-C vector), were co-transfected into cells. Firefly and Renilla luciferase activities were measured using the Dual-Luciferase Reporter Gene Assay Kit (Beyotime). The relative luciferase activity was calculated by normalizing the values of Firefly/Renilla.

### 4.7. Animal Experiments

Female BALB/c nude mice (5 weeks old) were obtained from Changsheng biotechnology, following the guidelines approved by the Institutional Review Board of the College of Life and Health Science, Northeastern University. The mice were housed in a specific-pathogen-free (SPF) animal facility.

To establish the subcutaneous tumor-bearing model, CRC cells were suspended in PBS at a concentration of 2 × 10^7^ cells/mL and injected into the dorsal region of the BALB/c nude mice (0.15 mL per site/animal; *n* = 6). Tumor volumes were calculated using the formula W^2^ × L/2, where W represented the short axis and L represented the long axis. The mice were euthanized, and the tumors were analyzed when the tumor volume reached approximately 1000 mm^3^.

To establish the experimental lung metastasis model, CRC cells were suspended in PBS at a concentration of 10^7^ cells/mL and injected into BALB/c nude mice via a tail vein injection (0.10 mL per site/animal; *n* = 5) to induce lung metastases. After four weeks, the mice were euthanized, and their lungs were subsequently removed for examination.

### 4.8. Hematoxylin–Eosin Staining

Hematoxylin–eosin staining can be used to identify the morphological features of metastatic tumors. Histological features like variable-sized cells, an increased nuclear plasma ratio, an extended mitotic phase, and visible nucleoli are frequently observed in tumors.

The excised tissues were fixed in 4% paraformaldehyde, embedded in paraffin, and sectioned at a thickness of 5 μm. The tissue sections were then dewaxed in xylene and rehydrated using a series of graded alcohols. The stained sections were processed for hematoxylin and eosin staining following the manufacturer’s guidelines. Finally, the staining was observed and captured using an Olympus microscope (Olympus, Tokyo, Japan).

### 4.9. Statistical Analysis

The miRNA sequencing data for the miRNA expression profiles (BioProject ID: PRJNA884845) used were registered with the National Center for Biotechnology Information BioProject database. All basic statistical charts were created using Graphpad Prism 7 software, all image analysis was performed using ImageJ software (NIH), and all experimental data were analyzed using SPSS statistical software (IBM SPSS Statistics 22.0, IBM, Amenk, New York, USA). The data between two groups were compared using the unpaired two-tailed Student’s *t*-test, and group differences were assessed using a one-way analysis of variance (ANOVA). Statistical significance was defined as * *p* < 0.05, ** *p* < 0.01, *** *p* < 0.001, and ns indicated no statistical significance.

## Data Availability

The data that support the findings of this study are available from the corresponding author upon reasonable request.

## Supplementary Materials

The following supporting information can be downloaded at: https://www.mdpi.com/article/10.3390/ijms242316740/s1.
