# Supplementary material for: BAP31-Mediated miR-206/133b Cluster Promotes Transendothelial Migration and Metastasis of Colorectal Cancer
Source: Int J Mol Sci. 2023 Nov 25;24(23):16740. doi: 10.3390/ijms242316740 (PMC10706076; doi:10.3390/ijms242316740)
Supplement: Supplementary file 1 [file ijms-24-16740-s001.zip › Supplementary material-Figures.pdf]

Supplementary Figures

Fig. S1

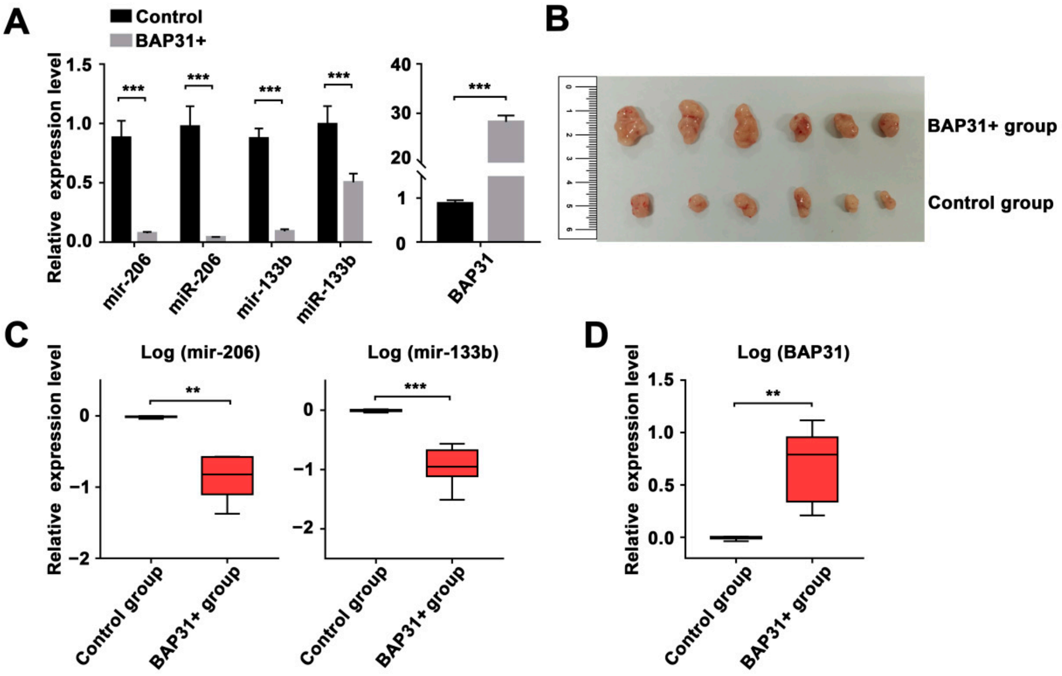

(**A**) qPCR analyzed the expression of miR-206/133b cluster precursors and matures in SW480 cells with BAP31 overexpression. (**B**) Xenograft tumors of BAP31-overexpressing group and Control group in nude mice.(**C**) qPCR analyzed the expression of miR-206/133b cluster precursors in CRC xenograft tumors. (**D**) qPCR analyzed BAP31 expression in CRC xenograft tumors. Data are represented as the mean ± SD of three independent experiments. ** $p < 0.01$, and *** $p < 0.001$.

Fig. S2

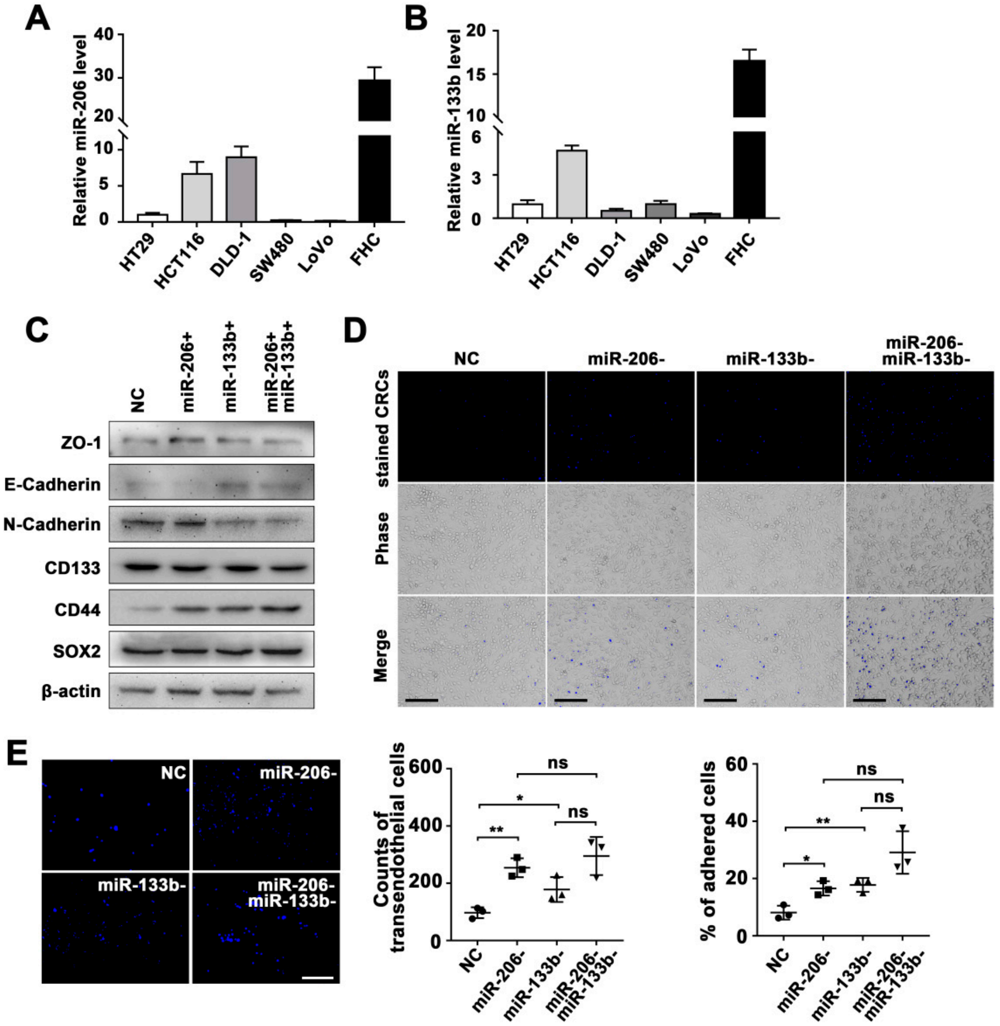

(**A, B**) qPCR analyzed the expression of miR-206 (A) and miR-133b (B) in CRC cells. (**C**) Western blot analyzed related proteins expression, reflecting the effect of miR-206 and miR-133b in LoVo cells. (**D**) Adhesion assays analyzed the effect of miR-206 and miR-133b in LoVo cells. The representative microscopic images demonstrate the phase contrast of Hoechst33342-stained HCT116 cells remaining on the layer of HUVECs after multiple washes (Scale bar, 100 μm). (**E**) Transendothelial migration assays analyzed the effect of miR-206 and miR-133b in LoVo cells. The representative microscopic images demonstrate the phase contrast of the Hoechst33342-stained HCT116 cells (blue) during transmigration through an endothelial barrier (Scale bar, 50 μm). Data are represented as the mean ± SD of three independent experiments. * $p < 0.05$, ** $p < 0.01$, and ns indicates non-significance.

Fig. S3

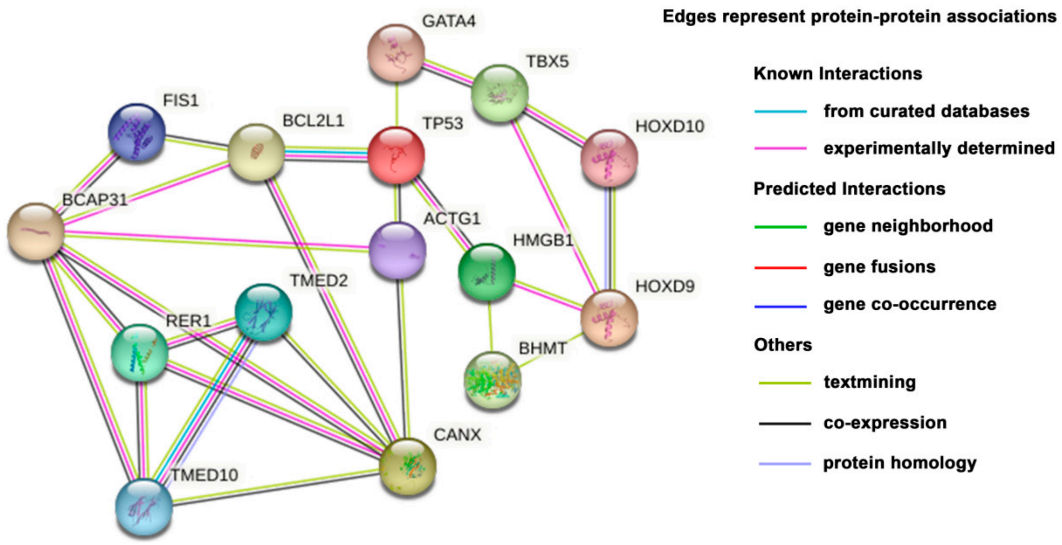

Schematic diagram depicted protein interaction analysis of BAP31 gene with HOXD9 and HOXD10 gene by STRING database.

Fig. S4

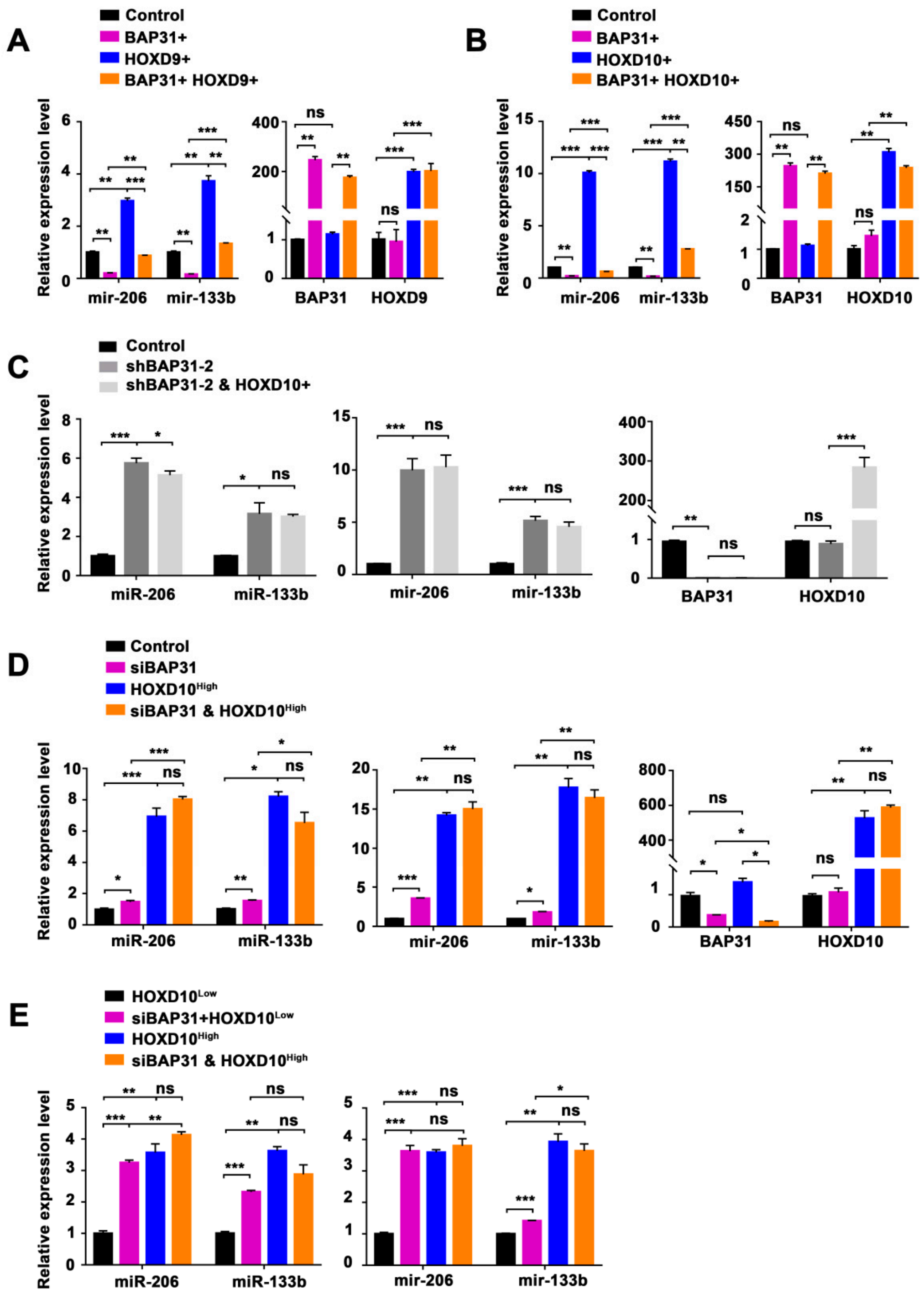

(**A**) qPCR analyzed the expression of miR-206/133b cluster precursors (left panel), reflecting the effect of BAP31 and HOXD9 (right panel) in HCT116 cells. (**B**) qPCR analyzed the expression of miR-206/133b cluste precursors (left panel), reflecting the effect of BAP31 and HOXD10 (right panel) in HCT116 cells. (**C**, **D**, **E**) qPCR analyzed the expression of miR-206/133b cluster matures and precursors, reflecting the effect of BAP31 and HOXD10 in HCT116 cells. Data are represented as the mean ± SD of three independent experiments. * $p < 0.05$, ** $p < 0.01$, *** $p < 0.001$, and ns indicates non-significance.
