# Supplementary material for: BAP31-Mediated miR-206/133b Cluster Promotes Transendothelial Migration and Metastasis of Colorectal Cancer
Source: Int J Mol Sci. 2023 Nov 25;24(23):16740. doi: 10.3390/ijms242316740 (PMC10706076; doi:10.3390/ijms242316740)
Supplement: Supplementary file 1 [file ijms-24-16740-s001.zip › Supplementary material-Tables.pdf]

Supplementary Tables

Table S1 List of miR-206/133b cluster and miR-133a-1/1-2 cluster

	1	2	3	4	5
mature miR_name	miR-206	miR-133b	miR-1-3p	miR-133a-5p	miR-133a-3p
Accession	MIMAT0000462	MIMAT0000770	MIMAT0000416	MIMAT0026478	MIMAT0000427
pre-mir name	mir-206	mir-133b	mir-1-2	mir-133a-1	mir-133a-1
stem loop-Accession	MI0000490	MI0000822	MI0000437	MI0000450	MI0000450
Genome context	chr6 52,144,349-52,144,434[+]	chr6 52,148,923-52149041[+]	chr18 21829004-21829088[-]	chr18 21825698-21825785[-]	chr18 21825698-21825785[-]
Position	Intergenic	Intergenic	host gene	host gene	host gene
raw_Ctrl	1909	489	1923	192	——
raw_BAP31+	1632.333333	394	1688.833333	160.5	——
norm_Ctrl	2009.198257	514.66629	2023.933079	202.0775617	——
norm_BAP31+	1434.022449	346.1332519	1483.658309	141.0009821	——
fold_change (BAP31+/Ctrl)	0.713728695	0.672539194	0.733056999	0.697756747	——
p value (fisher_test)	4.48172e-38	5.87584e-13	1.87867e-34	0.0000286157	——
p value (chi_square_22)	4.0019e-38	6.1256e-13	1.7765e-34	0.000026785	——

Table S2 List of Real-time PCR primer sequences

Primer name	Sequence (5'—3')
GAPDH Forward	GACAGTCAGCCGCATCTTCT
GAPDH Reverse	TTAAAAGCAGCCCTGGTGAC
BAP31 Forward	TTGCTGCTGTCCTTCCTGCTTAGA
BAP31 Reverse	ATGTACTTCTTGGCCGCCTCACTA
CDC42 Forward	CTTAAGGGGAGGAGGACGGA
CDC42 Reverse	GAGCACCAGATGGGGAACAT
ARPC5 Forward	GAGCCCGTCTGACAATAGCA
ARPC5 Reverse	GCGGTGGCATTTGGTTGTTT
HOXD9 Forward	GGCAGCGGATTTGTTGTTGT
HOXD9 Reverse	TTTACAACTGGTCCTCGGGC
HOXD10 Forward	GTTAACCTCACCGACAGGCA
HOXD10 Reverse	ACAGATCACTTGCAGCACGA
mir-206 Forward	CTTCCCGAGGCCACATGCTT
mir-206 Reverse	CACTTGCCGAAACCACACACT
mir-133b Forward	GAAAGATGCCCCCTGCTCTG
mir-133b Reverse	TGGTTGAAGGGGACCAAACC
U6 Forward	CTCGCTTCGGCAGCACA
U6 Reverse	AACGCTTCACGAATTTGCGT
miRNAs Reverse	CTCAACTGGTGTCGTGGA
cDNA-miR-206	CTCAACTGGTGTCGTGGAGTCGGCAATTCAGTTGAGCCACACA
miR-206 Forward	GCCGAGTGGAATGTAAGGAA
cDNA-miR-133b	CTCAACTGGTGTCGTGGAGTCGGCAATTCAGTTGAGTAGCTGG
miR-133b Forward	GCCGAGTTTGGTCCCCTTCA
cDNA-miR-1-3p	CTCAACTGGTGTCGTGGAGTCGGCAATTCAGTTGAGATACATAC
miR-1-3p Forward	GCCGAGTGGAATGTAAAGAA
cDNA-miR-133a-5p	CTCAACTGGTGTCGTGGAGTCGGCAATTCAGTTGAGATTTGGT
miR-133a-5p Forward	GCCGAGAGCTGGTAAAATGG
cDNA-miR-133a-3p	CTCAACTGGTGTCGTGGAGTCGGCAATTCAGTTGAGCAGCTGG
miR-133a-3p Forward	GCCGAGTTTGGTCCCCTTCA

Table S3 List of the scores of miRNA target-predicting algorithms

miR name	Genes	miRDB	TargetMiner				TargetScan		miRanda	
		Target Score	6mer	7mer -A1	7mer -m8	8mer	Total context++ score	Aggregate PCT	Tot Score	Tot Energy
miR-133b	DLG2	54	7	2	1	1	——	——	140	-18.3
	RAP2C	85	1	1	——	1	——	——	152	-17.06
	PPP2CB	95	——	——	——	——	-0.63	0.87	140	-12.05
miR-206	ARPC5	83	——	——	——	——	-0.32	0.69	129	-17.86
	PRKACB	81	5	2	1	——	-0.28	0.72	147	-8.48
	PRKCE	87	7	——	1	1	-0.22	0.34	145	-13.17
	AMOT	67	7	2	2	——	-0.14	0.59	158	-16.65
	DLG2	74	11	1	——	——	-0.14	0.65	131	-19
	CDC42	63	——	——	——	——	-0.28	0.17	147	-18.7
	PRKCI	57	4	2	——	——	——	——	140	-15.55
	NEDD4L	53	1	——	——	1	——	——	146	-20.22
	CCND1	60	8	2	1	——	——	——	143	-15.17

Table S4 List of the cis-acting regulatory DNA elements (TATA-boxes, CAAT-boxes, and E-boxes) of three reg ions containing potential TSSs of the miR-206/133b cluster

region	range	Sequence	Num.	Loc.(Str.)
T-1	EBOXBNNAPA	CANNTG	8	52,136,523, 52,136,933, 52,137,157, 52,137,187, 52,137,245, 52,137,278, 52,137,308, 52,137,322
T-1	CAATBOX1	CAAT	4	52,136,563, 52,136,634, 52,136,750, 52,137,157
T-2	EBOXBNNAPA	CANNTG	5	52,138,989, 52,139,452, 52,139,614, 52,139,826, 52,139,953
T-2	CAATBOX1	CAAT	1	52,139,004
T-2	TATABOX3	TATTAAT	1	52,139,763
T-2	TATABOX5	TTATTT	2	52,139,416, 52,139,631
T-3	EBOXBNNAPA	CANNTG	11	52,141,310, 52,141,359, 52,141,420, 52,141,715, 52,141,830, 52,141,832, 52,141,873, 52,141,887, 52,141,974, 52,141,982, 52,142,092
T-3	CAATBOX1	CAAT	5	52,141,402, 52,141,477, 52,141,765, 52,141,974, 52,142,092
T-3	TATABOX3	TATTAAT	1	52,141,188
T-3	TATABOX5	TTATTT	1	52,141,222

Table S5 List of the binding sites of potential transcription factors in the T-3 region

TF name	Binding position	Dissimilarity	String	RE equally	RE query
HOXD9	22-31	7.030183	AATAATAGTA	0.04204	0.14279
HOXD9	25-34	11.797151	AATAGTAATA	0.02522	0.09583
HOXD9	56-65	14.123515	AATATTATGC	0.08408	0.16809
HOXD9	601-610	7.270719	ATTTTATATT	0.03783	0.18059
HOXD9	705-714	9.979471	AACTGCTATT	0.12822	0.48371
HOXD9	775-784	11.174228	AATATCACCC	0.10299	0.24952
HOXD10	22-31	7.030183	AATAATAGTA	0.04204	0.14279
HOXD10	25-34	11.797151	AATAGTAATA	0.02522	0.09583
HOXD10	56-65	14.123515	AATATTATGC	0.08408	0.16809
HOXD10	601-610	7.270719	ATTTTATATT	0.03783	0.18059
HOXD10	705-714	9.979471	AACTGCTATT	0.12822	0.48371
HOXD10	775-784	11.174228	AATATCACCC	0.10299	0.24952
c-Fos	355-364	5.119614	GAGTCATTTA	0.01261	0.01463
RAR-β	521-530	7.47824	AGGGTTTTGA	0.13452	0.09053
RAR-β	668-677	7.496706	CTTCAACCCA	0.13452	0.09053
RAR-β	695-704	10.667372	GGGGTTACAC	0.07567	0.05187
RAR-β	895-904	8.541284	AAAAAACCCA	0.14713	0.0962
NF-AT2	821-830	2.800326	AATTCTTTCC	0.01892	0.03351
NF-AT2	891-900	9.755755	GGAAAAAAAA	0.04834	0.08087
NF-AT2	1033-1042	9.755755	TTGTTTTTCC	0.04834	0.08087
NF-AT1	822-831	5.512555	ATTCTTTCCA	0.03258	0.04107
NF-AT1	890-899	4.823485	TGGAAAAAAA	0.04204	0.05578
NF-AT1	1034-1043	4.134416	TGTTTTTCCA	0.04624	0.06432
STAT1β	825-834	9.807397	CTTTCCATTA	0.08197	0.09722
STAT1β	887-896	14.492168	ATCTGGAAAA	0.1135	0.12515
TBP	603-612	1.871542	TTTATATTGC	0.10089	0.37873
MEF-2A	49-59	13.303905	AACTTCAAATA	0.04414	0.16622
MEF-2A	711-721	13.303905	TATTTACAGAT	0.04414	0.16622
MEF-2A	876-886	14.541822	CTAGATAAATA	0.13058	0.39206
MEF-2A	993-1003	2.660781	TATTTTTGTCT	0.00315	0.01193
POU2F1	933-943	13.168602	CCTATGCAAAG	0.12611	0.25387
CTF	215-226	2.10785	TAGCCAATAACC	0.00053	0.00047
CTF	610-621	12.50062	TGCCATTGGCCC	0.01419	0.00594
CTF	789-800	14.60847	CACAATTGGCCA	0.03783	0.03832
GATA-3	864-875	14.461588	GTAGATAGGAAA	0.02115	0.02781

Table S6 List of specification

Name	Specification
Genes	
CDC42	Cell division cycle 42 (CDC42) is a small GTPase of the Rho-subfamily, which regulates signaling pathways that control diverse cellular functions including cell morphology, migration, endocytosis and cell cycle progression. This protein could regulate actin polymerization through its direct binding to Neural Wiskott-Aldrich syndrome protein (N-WASP), which subsequently activates Arp2/3 complex.
ARPC5	Actin related protein 2/3 complex subunit 5 (ARPC5) is a gene that codes for one of the human Arp2/3 protein complex's seven subunits. The Arp2/3 protein complex has been implicated in the control of actin polymerization in cells.
HOXD9	Homeobox D9 (HOXD9) is a gene that is a member of the homeobox gene family. In all multicellular organisms, the homeobox genes encode a family of transcription factors that are highly conserved and essential to morphogenesis.
HOXD10	Homeobox D10 (HOXD10) is a gene that belongs to the Abd-B homeobox family and that produces a protein that has a DNA-binding homeobox domain. Involved in limb development and differentiation, the encoded nuclear protein is expressed in the developing limb buds and acts as a sequence-specific transcription factor.
Cells	
HUVEC	HUVEC [HUV-EC-C] is an endothelial cell line that was isolated from the vein of the umbilical cord.
HCT116	HCT116 is a colorectal cell line that was isolated from the colon of an adult male with colon cancer.
DLD-1	DLD-1 is a colorectal adenocarcinoma cell line that was isolated from the large intestine of a colon adenocarcinoma patient.
SW480	SW480 [SW-480] is a colorectal adenocarcinoma cell line that was isolated from the large intestine of a Dukes C colorectal cancer patient.
HT29	HT29 [HT-29] is a colorectal adenocarcinoma cell line that was isolated in 1964 from a primary tumor obtained from a 44-year-old, White, female patient with colorectal adenocarcinoma.
LoVo	LoVo is a colorectal adenocarcinoma cell line that was isolated in 1971 from the large intestine of a White, 56-year-old, male with grade IV Dukes C colorectal cancer patient.
HEK-293T	HEK-293T [293T] is an epithelial-like cell line that was isolated from the kidney of a patient.

Table S7 List of antibodies

Names	Manufacturer	Concentration
Primary antibodies		
β-actin Mouse mAb	CST	1:1000
BAP31 Rabbit mAb	Sigma	1:500 - 1:2000
MMP2 Rabbit mAb	Proteintech	1:500 - 1:1000
VEGFA Rabbit mAb	Wanlei	1:1000 - 1:2000
CDC42 Rabbit mAb	Proteintech	1:500 - 1:2000
ARPC5 Rabbit mAb	Proteintech	1:500 - 1:1000
HOXD10 Rabbit mAb	Abcam	1:1000 - 1:10000
Lamin A/C Mouse mAb	CST	1:2000
ZO-1 Rabbit mAb	Wanlei	1:1000 - 1:2000
E-Cadherin Rabbit mAb	Wanlei	1:1000 - 1:2000
N-Cadherin Rabbit mAb	Wanlei	1:1000 - 1:2000
CD133 Rabbit mAb	Wanlei	1:500 - 1:1000
CD44 Rabbit mAb	Wanlei	1:300 - 1:1500
SOX2 Rabbit mAb	Wanlei	1:1000 - 1:1500
Secondary antibodies		
Anti-rabbit IgG, HRP-linked Antibody	CST	1:1000 - 1:3000
Anti-mouse IgG, HRP-linked Antibody	CST	1:1000 - 1:3000
